# Knowledge, and practices on sexual and reproductive health among youth trainees attached to youth training centers in Sri Lanka

**DOI:** 10.1186/s40834-023-00216-0

**Published:** 2023-03-02

**Authors:** D. Mataraarachchi, P. V. S. C. Vithana, A. Lokubalasooriya, C. J. Jayasundara, A. S. Suranutha, T. E. A. Pathirana, C. De Silva

**Affiliations:** 1 Family Health Bureau, Colombo, Sri Lanka; 2 Base Hospital, Panadura, Sri Lanka

**Keywords:** Adolescents, Sexual and reproductive health, Teenage pregnancy, Sexually transmitted disease, Family planning

## Abstract

**Background:**

Sexual and reproductive health is a major concern among adolescents and youth in Sri Lanka. The study was carried out to assess the knowledge, and practices of the youth trainees attached to youth training institutes in Sri Lanka.

**Methodology:**

A descriptive, cross-sectional study was carried out among randomly selected 425 youth trainees attached to youth training centers in Sri Lanka using a pre-tested self-administered questionnaire. Statistical analysis was conducted using SPSS-21. Categorical variables were presented as numbers and percentages. A comparison of categorical variables was conducted using the Chi-Square test and Fisher’s exact test as applicable. The bivariate logistic regression model was used to determine the independent association of the selected demographic factors with youth trainees’ sexual and reproductive health knowledge.

**Results:**

Study group consisted of 51.8% (*n* = 220) males and 48.2% (*n* = 205) females with a mean age of 18.6 years (SD = 1.8). Youth trainees’ knowledge of the physiology of the reproductive tract was not at a satisfactory level, where Only 49% (*n* = 211) knew that nocturnal emission is normal in young men. The trainee’s knowledge of contraception was also at a poor level. Only 47.5% (*n* = 202) had ever heard of condoms, and 13.2% (*n* = 56) knew about the emergency contraceptive pill. Nearly 8% (*n* = 33) of the youth had engaged in sexual intercourse at least once in their lifetime. Male gender (AOR = 2.3, *p* < 0.001), and age above 20 years (AOR = 1.9, *P* = 0.005) were positively associated with SRH knowledge.

**Conclusions and recommendations:**

Knowledge and practices on sexual and reproductive health among youth trainees were sub-standard. The study recommends strengthening sexual and reproductive health education at youth training centers.

## Introduction

### Global situation

Improving Sexual and Reproductive Health (SRH) in adolescent girls is one of the primary concerns in sustainable development goals (2015–2030). Reproductive health is primarily included in goals 3 and goal 5. Target 3.7 states about ensuring universal access to reproductive health care services that include family planning services, information and education, and integration of reproductive health into national strategies and programs [1]. Global evidence indicates that each year nearly sixteen million girls aged 15–19 around the globe give birth, which accounts for 11% of all births worldwide. Approximately 95% of these births occur in low and middle-income countries [2]. Young persons below the age of 20 years face a higher risk of complications and deaths as a result of pregnancy than women aged above 20, while their babies face a higher risk of low birth weight, preterm delivery, and severe neonatal complications [3]. Moreover, sexually transmitted diseases are becoming a growing concern among adolescents and youth across the world. Globally, an estimated number of 410,000 young persons in the age group of 10–24 years were newly infected with HIV in the year 2020, of whom 150,000 were adolescents [4].

### The situation in Sri Lanka

Nearly one-fourth of the Sri Lankan population is comprised of adolescents and youth aged 10–24. This is an important group of the country’s population that will decide the country’s financial and political stability of in the future. Hence, investing in the health and well-being of adolescents and youth is crucial for ensuring a better tomorrow.

Evidence shows that Sexual and Reproductive Health issues have become one of the major concerns in Sri Lankan adolescents and youth [5]. The World Bank reports indicate that the adolescent fertility rate has been static without much improvement over the past decade where births per thousand adolescents aged 15–19 years was 19.5 in the year 2010, while it was 20.5 in 2020 [6]. National HIV/AIDs prevalence in Sri Lanka is 0.01%. In the period between 2011 and 2018, the number of HIV-infected persons has increased from 78 to 285 in men, while 65 cases were annually reported among women [7]. The data indicates that the new infection increases especially among young people [8, 9], where out of the 600 HIV cases reported to the National STI and AIDs Control Program in 2022, 150 are youth in the age group of 18-30 years [1]. In addition to that, it is also reported that nearly 14% of both male and female schooling adolescents in Sri Lanka aged 10–19 years face some form of sexual abuse [10].

National youth health survey assessed certain components of sexual and reproductive health knowledge and practices among youth as a whole, and as school-going and non-school-going categories in 2012 [11]. The survey demonstrated that nearly 50% of the Sri Lankan youth are unaware of common SRH issues. It was also found that 14.7% of the Sri Lankan youth are sexually active, while the majority of them were out of school. Further, among sexually active youth, only 30.4% reported using condoms during the preceding year [11].

Over the years reproductive health interventions were carried out targeting school children in Sri Lanka. This included the incorporation of the sexual and reproductive health module into the school curriculum and community-based interventions. The sexual and reproductive health module is delivered to school children as a part of the Health Science curriculum by the teacher responsible while the community-based sexual health interventions are planned and conducted in the field through the area Medical Officer of the Health office. However, researchers have indicated several gaps in the existing school-based and community-based sex education programs [12, 13]. The National Youth Health Survey 2012/13 [14] showed that only 59% of the youth received SRH education in schools. In a study that assessed sexual and reproductive health knowledge, attitudes, and practices among Sri Lankan adolescents, only less than 1% of adolescents were found to have satisfactory SRH levels [14]. In a survey carried out in the plantation sector, youth considered teachers as the most unreliable source of SRH information [8]. A study conducted among a nationally representative sample of first-year university students in Sri Lanka in 2018 indicated that the majority (80.6%) did not receive adequate sexual and reproductive health education from school, while only one-fifth (19.6%) were adequately knowledgeable about contraceptive methods [15]. The same study revealed that the “school teacher” was the least important source of sexual health information to children. Qualitative interviews with the school teachers revealed that teachers were shy to deliver sexual health information. Furthermore, teachers believed that “having sex is wrong (for students)”, “syllabus should be updated”, and “they should be taught about safe sex rather than physiology” [15]. Further to this, a study carried out in Kandy, Sri Lanka found that those who reported of not receiving sex education in school were nearly twice as more likely to have self-poisoned than those who did (OR = 1.68, 95% CI: 1.1–2.5) [16].

The current practice of delivering SRH education in schools in Sri Lanka neglects the out-of-school youth or the most vulnerable population who are more at risk of developing sexual and reproductive health issues. Since youth training institutes give shelter to both the schooling and out-of-school youth in the country, these institutes were identified as a better platform to deliver SRH knowledge to the young generation in Sri Lanka.

Youth training institutes were established under the National youth service council and National Youth Corps in Sri Lanka to develop a skillful young generation in the country. These centers provide opportunities for youth aged 15–29 years around the country in a wide range of disciplines including soft skills and advanced skills. Currently, there are 49 youth training centers under the purview of the National Youth Corps, in Sri Lanka and 52 centers under the purview of the National Youth Service Council.

As a collaborative effort of the Family Health Bureau and the Ministry of Youth affairs Sexual and Reproductive health module was introduced to the youth training center curriculum in the year 2018, to improve youth trainees’ knowledge of SRH. The module consisted of theory and practical sessions that covers the main SRH topics; An introduction to sexual and reproductive health, landmarks in human reproduction, pregnancy, abortion, and contraception, prevention of sexually transmitted diseases, sexual health promotion, responsible behavior in SRH, life-skill-based decision-making in SRH, and available health and non-health services. The module consisted of ten lectures, group discussions, and brainstorming sessions. The total duration of the module was 18 hours, while each session lasted for nearly 45 minutes to 1.5 hours. The module was taught by the instructors attached to youth training centers who were trained by the Family Health Bureau, the focal point of adolescent and youth health. The instructors were trained by medical officers who have specialized knowledge in adolescent and youth health as well as adolescent sexual and reproductive health. A three-day residential training program was conducted to train the instructors where they were given the opportunity to clarify any queries.

The intervention was successfully implemented in 20 training centers under the youth corps and National youth service council as a pilot project. Assessing the effectiveness of the newly introduced module in improving youth knowledge of SRH was required to decide on further scaling up of the intervention.

This research was carried out to assess the knowledge, and practices on sexual and reproductive health matters among youth trainees attached to youth training institutes in Sri Lanka, in which the sexual and reproductive health module is successfully being implemented at present. Although, the WHO definition of youth falls between 10 and 24 years, since youth training centers in Sri Lanka gave shelter to young persons aged 15–29 years, and the objective of the study focused on all youth trainees attached to the youth training centers, the sample consisted of persons in the particular age range.

The present study’s findings will help identify the gaps in the current training curriculum and provide guidance to do necessary revisions to the existing SRH module in the training curriculums.

## Methodology

A descriptive, cross-sectional study was carried out among 425 youth trainees aged 15–29 years attached to training centers of Youth Corps and National Youth Service Council which are functioning under the Ministry of Youth Affairs in Sri Lanka, to assess their knowledge, attitudes, and practices on sexual and reproductive health.

The sample of youth trainees was selected using a two-stage cluster sampling method. Six youth training centers were selected using computer-generated random numbers after listing out all the centers in which the SRH module was incorporated into the curriculum. From each center, an equal number of study subjects were selected using simple random sampling techniques. The sample of trainees were selected after listing out all eligible youth trainees who have been following a training course in a youth training center for more than 6 months. Since 80 % attendance in all modules was obligatory to sit the final examination, all trainees were included in the sample assuming that they all have 80% attendance to the module. Course attendance details were obtained from the training instructor in charge of the module. Trainees who have been following a course for less than 6 months period and those who were suffering from severe physical or mental disabilities which prevented them from attending lectures during the time of data collection or those who did not have 80% attendance to the module were excluded from the study.

The sample size was computed using the standard formula [17]. The anticipated proportion of trainees having satisfactory sexual and reproductive health knowledge was taken as 50% since we couldn’t come across a previous study conducted in a similar study setting.

A self-administered questionnaire that was developed with an extensive literature review and expert opinion was used for data gathering. The questionnaire contained questions that were included in the National Youth Health Survey [13] and in other Sri Lankan studies that assessed SRH knowledge and practices among young persons [18]. An expert panel consisted of three public health experts, two MOHs, and two youths.

The developed questionnaire was pretested among a sample of 30 youth in the same age group selected from a different youth training center. The questionnaire included questions to assess the trainee’s knowledge of male and female reproductive tract and function, family planning, pregnancy, abortion, and sexually transmitted diseases. The questionnaire also assessed the trainee’s sexual and reproductive health practices. Following the development of the questionnaire, it was tested for face, content, and consensual validity by a panel of experts using a modified Delphi technique. The Cronbach’s alpha value > 0.6 indicated fair alignment of the questions with each other. The correlation coefficient for test-retest reliability above 0.7 indicated good agreement between the responses.

Data collection was done by trained youth who were awaiting university entrance. Data collectors visited each training center to collect data from youth trainees. Data collection was conducted with minimal disturbance to the academic activities. Informed, written consent was obtained from all eligible participants before the distribution of the questionnaires.

All data were coded and entered into a database that had been created using the standard statistical package SPSS-21. Categorical variables were presented as numbers and percentages. The bivariate logistic regression model was used to determine the independent association of the selected demographic factors with youth trainees’ sexual and reproductive health knowledge. Ethical clearance was obtained from the Faculty of Medicine, University of Colombo, while administrative clearance was obtained from the Ministry of Youth Affairs and the authorities at youth training centers.

## Results

Four hundred and twenty-five youth trainees participated in the assessment. The response rate was 98%.

### Socio-demographic variables

Over 85% of the group were Sinhalese (*n* = 365; 85.9%) with 51.8% (*n* = 220) of the group consisting of males and 48.2% (*n* = 205) being females. The age of the group ranged from 17 to 29 years with a mean age of 18.6 years with a standard deviation of 1.8 years.

Over 80% (*n* = 351; 82.6%) of the sample were Buddhists. Ninety-six percent (*n* = 406) were educated at school up to grade 11 or above while all had an education level of grade 6 and above. Of the group, only 4% (*n* = 17) were part-time employed. Among the sample, only 5 (1.2%) were married. Out of the sample, eighty (18.9%) were staying at a place apart from their own house.

Nearly half of the group (*n* = 226, 55.7%) expressed that their family income per month was Rs.20000.00 or below, which was below the average household income in Sri Lanka, which was around 75,000 during this time before the country fell into a deep economic recession [19]. Over 90% (92%, *n* = 391) of the trainees in the sample were following a 6-month course. Only 7.3% (*n* = 31) were following longer courses than 6 months (Table 1).

**Table 1:** Distribution of Youth Trainees by Socio-Demographic Characteristics

Variable	Number (n)	Percentage (%)
**Nationality (*n*** **= 425)**		
Sinhala	365	85.9
Tamil	26	6.1
Muslim	32	7.5
Others	2	0.5
**Age in Years (*n*** **= 425)**		
Mean	18.6	SD = 1.821
**Sex (*n*** **= 425)**		
Female	205	48.2
Male	220	51.8
**Religion (*n*** **= 425)**		
Buddhist	351	82.6
Christion	22	5.2
Hindu	20	4.7
Islam	32	7.5
**Highest Education Level (*n*** **= 421)**		
Grade 6–10	15	3.6
Grade 11 and above	406	96.4
**Marital Status (*n*** **= 414)**		
Never married	409	98.8
Married or living together	5	1.2
**Place of living (*n*** **= 406)**		
Own home	344	81.1
Another place	80	18.9
**Family income (*n*** **= 406)**		
Rs.20000 and below	226	55.7
> Rs.20,000	180	44.3
**Having siblings (*n*=425)**		
Yes	386	90.8
No	39	9.2
**Duration of training (*n*** **= 422)**		
6 months and below	391	92.7
> 6 months	31	7.3

### Knowledge of Sexual & Reproductive Health

Only 309 (73.7%) knew that the sperms are produced by the testicles, while 48.2% (*n* = 203) incorrectly believed that ova are produced by the uterus. Only 41.3% (*n* = 174) knew that testosterone is a male hormone responsible for male pubertal changes. Nearly 48% (*n* = 204) believed that size of the sexual organs can affect their reproductive capability (Table 2).

**Table 2:** Youth Trainee’s Knowledge of Sexual Changes during Puberty and the Physiology of the Reproductive Tract

Variable	Number (n)	Percentage (%)
**The start of a new life begins with the fusion of sperm with an ovum (*n*** **= 421)**		
True	351	83.4
False	14	3.3
Do not know	56	13.3
**Sperm are produced in testis (*N*** **= 421)**		
True	309	73.4
False	10	2.4
Do not know	102	24.2
**Ova are produced by the uterus (*n*** **= 421)**		
True	203	48.2
False	121	28.7
Do not know	97	23.0
**Testosterone is a male hormone responsible for male pubertal changes (*n*** **= 421)**		
True	174	41.3
False	23	5.5
Do not know	224	53.2
**It is normal for adolescent males to have nocturnal emissions (*n*** **= 421)**		
True	211	50.1
False	31	7.4
Do not know	179	42.5
**he size of the penis and testicles can vary from one person to another (*n*** **= 421)**		
True	221	52.5
False	8	1.9
Do not know	192	45.6
**The size of the reproductive organ can affect a person’s reproductive capability (*n*** **= 421)**		
True	204	48
False	196	46.5
Do not know	21	49.9

### Trainee’s knowledge of family planning method

Only 35.5% (*n* = 151) of the youth claimed that they have never heard of family planning methods. The percentage of youth who had ever heard of condoms was 47.5% (*n* = 202), while only 13.2% (*n* = 56) knew about the emergency contraceptive pill (Table 3).

**Table 3:** Distribution of Youth Trainees by Knowledge of Family planning

Variable	Number (n)	Percentage (%)
**Heard about Depo-Provera injections (*n*** **= 421)**		
Yes	13	3.1
No	408	96.9
**Heard about an intrauterine device (*n*** **= 421)**		
Yes	28	6.7
No	393	93.3
**Heard about hormonal implant (*n*** **= 421)**		
Yes	13	3.1
No	408	96.9
**Heard about emergency contraceptive pills (*n*** **= 421)**		
Yes	56	13.3
No	365	86.7
**A condom can prevent getting sexually transmitted diseases (*n*** **= 421)**		
Yes	51	12.1
No	370	87.9

### Trainee’s knowledge of pregnancy, abortion, and sexually transmitted disease

Out of the group, 53.9% (*n* = 227) knew that teenage pregnancy can give rise to complications in both mother and baby.

Nearly 75 % (*n* = 318) of the youth knew that HIV/AIDs can be transmitted through unprotected sexual intercourse. Only 12.9% (*n* = 55) knew that by using condoms one can avoid contracting sexually transmitted diseases. The percentage of youth who incorrectly identified avoiding common toilet use or not sharing face towels as ways of preventing STI s were 6% (*n* = 26) and 19% (*n* = 81) respectively (Table 4).

**Table 4:** Distribution of Youth Trainee by Knowledge of Pregnancy, Abortion, and Prevention of Sexually Transmitted Infection

Variable	Number (n)	Percentage (%)
**Induce abortion may even lead to death (*n*** **= 421)**		
Yes	98	23.3
No	6	1.4
Do not Know	317	75.3
**Teenage pregnancy leads to complications for the baby and mother (*n*** **= 421)**		
True	227	53.9
False	16	3.8
Do not know	178	42.3
**HIV/AIDS can be transmitted by sexual intercourse (*n*** **= 421)**		
True	318	75.5
False	11	2.6
Do not know	92	21.9
**People can protect themselves from HIV/AID by using condoms corrected with every sexual intercourse (*n*** **= 421)**		
True	148	35.2
False	64	15.2
Do not know	209	49.6
**A person can prevent getting STI by abstaining from sex (*n*** **= 425)**		
Yes	114	26.8
No	311	73.2
**A person can prevent from getting infected with STI by using a condom when having sex (*n*** **= 425)**		
Yes	113	26.6
No	312	73.4
**A person can prevent from getting infected with STI by not using a common toilet (*n*** **= 425)**		
Yes	26	6.1
No	399	93.9
**A person can prevent getting SIT by not sharing a face towel (*n*** **= 425)**		
Yes	81	19.1
No	344	80.9

### Practices related to sexual and reproductive health

Out of the sample, 22.8% (*n* = 97) of youth knew a friend who has experienced sexual intercourse. Among them, 56.6% (*n* = 77) had sexual intercourse with their partner, while 11.8% (*n* = 16) were with a commercial sex worker.

Nearly 8% (*n* = 33) of the youth had engaged in sexual intercourse at least once in their lifetime. Out of those who have had sexual intercourse, nearly 74.2% (*n* = 23) had had their first sexual contact at the age of 14–18 years (Table 5).

**Table 5:** Sexual and Reproductive Health Practices among Youth Trainees

Variable	Number (n)	Percentage (%)
**Do you know a friend who has ever engaged in sexual intercourse? (*n*** **= 421)**		
Yes	136	32.0
No	289	68.0
**With whom did he/she have sexual intercourse? (*n*** **= 97)**		
Partner	77	56.6
Friend	13	9.6
Commercial sex worker	16	11.8
Relative	3	2.2
Other	27	19.9
**Have you ever had sexual intercourse? (*n*** **= 425)**		
Yes	33	7.8
No	392	92.2
**At what age did you first have sex? (*n*** **= 33)**		
Less than 14 years	4	12.9
14–18 years	25	74.2
Above 18 years	4	12.9
**If yes, with whom did you have sex? (*n*** **= 33)**		
Girl or boyfriend	48	59.3
Another friend	2	0.6
Relative	5	2.5
Commercial sexual worker	2	29.6
Other	24	
**Did you use condoms during sexual activity? (*n*** **= 33)**		
Yes	19	58.0
No	14	42.0
**Have you ever used any contraceptive other than condoms, during sexual intercourse (*n*** **= 33)**		
Yes	8	24.2
No	25	75.8

Of the ones who had engaged in sexual intercourse, only 57.6% (*n* = 19) had used condoms when having sex 24.2% (*n* = 8) claimed that they ever used a contraceptive other than condoms during sexual contact (Table 5).

### Associations of sexual and reproductive health knowledge among youth trainees

Male gender (AOR = 2.3, *p* < 0.001), and youth trainees aged above 20 years (AOR = 1.9, *p* = 0.005), were positively associated with SRH knowledge (Table 6).

**Table 6:** Associations of Sexual and reproductive health knowledge among youth trainees

Variable	Knowledge score > 50		Knowledge score < 50		OR	OR	Significance(p)
	Number (N)	Percentage (%)	Number (N)	Percentage (%)	(Unadjusted)	(adjusted)	
**Sex**							
**Male**	61	27.7	159	72.3	**2.1**	**2.3**	
Female	22	10.7	183	89.3	**(1.3–3.3)**	**(1.4–3.6)**	**0.001**
**Age**							
15–20 years	59	20.8	225	79.2	**2.1**	**1.9**	
**Above 20 yrs**	43	35.5	78	64.5	**(1.3–3.3)**	**(1.2–3.2)**	**0.005**
**Religion**							
Buddhist	95	27.1	256	72.9	2.1	1.7	
**Other**	11	14.9	63	85.1	(1.1–5.0)	(0.8–3.5)	0.11
**Education level (*n*** **= 421)**							
Grade 6–10	15	100	0	0	4.9	3.4	0.24
**Above 11**	300	73.9	106	26.1	(0.6–38.7)	(0.43–27)	

## Discussion

Results indicated that youth trainees’ knowledge of physiological changes during puberty, pregnancy, conception, and family planning was not at a satisfactory level. Many youths in our study sample were not aware of the complications of teenage pregnancy (46%, *n* = 198) or the consequences of induced abortion (76.7%, *n* = 327). Moreover, the youth trainee’s knowledge about different contraceptive methods was alarming, and a very high percentage of youth had not even heard of available contraceptive methods. It was seen that the male gender, age above 20 years, being a Buddhist, and having an educational level above O/L were positive determinants of SRH knowledge.

Similar to the above findings National Youth Health survey 2012/13 has indicated that knowledge about SRH among young persons is not up to the expected level [8]. It is found that the main reason for poor knowledge of SRH is associated with the societal stigma on providing sexual health information to children [20]. Literature indicates that even though sexual health education has been a compulsory part of the Sri Lankan school curriculum for the past three or four decades, there are many gaps in providing SRH knowledge to children through a school-based curriculum [12]. Being a stigmatized and confidential topic in society, teachers not being confident enough to deliver the module, and shyness and embarrassment were the main reasons for the failure in delivering the SRH module to school children [21].

A major reason for poor knowledge among youth trainees on available contraceptive methods could be related to society’s preference for abstinence-only sex education over abstinence-plus sexuality education [12]. Studies have found that many adults including parents as well as teachers are against providing family planning information to children as they fear that this would promote sexual activity among youth [12].

We may attribute the above findings to the present study findings. Even though the SRH module was incorporated into the training curriculum in youth training centers for nearly 3 years, these could be the reasons behind the poor SRH knowledge among youth trainees. An assessment needs to be carried out among the students as well as the teacher instructors to explore how well the module content has been delivered to the students.

In the present study, only 7.8% of trainees admitted that they ever engaged in sexual intercourse. However, a study that was carried out in youth Corps centers in the Western province in 2020 showed that 15.5% of youth engaged in sexual activity and the majority of them (79.2%) were having multiple partners [22]. However, the findings of the National youth health survey indicated that nearly one-third of the Sri Lankan youth engaged in some form of sexual activity during the preceding year. Further, the National Survey on emerging issues indicated that among Sri Lankan youth aged 14–19 years, 6% engage in heterosexual activities while 10% are in homosexual relationships [21]. The difference in the findings can be linked to the fact that the data collection venue in the present study was youth training centers and youth trainees’ hesitation to provide true information due to fear and embarrassment. As per the above hypothesis, 32% of the youth in the present study responded as knowing a friend who has engaged in sexual intercourse. Similarly, in a qualitative study that was carried out among unmarried youth in Sri Lanka, most of the youth expressed that at least one of their friends is sexually active with their partner contrary to the conservative beliefs in society regarding premarital sex or virginity [23].

The results of the present study indicated that 58% of the youth who reported having engaged in sexual intercourse have used condoms as a contraceptive method, while 24.2% used another contraceptive other than condoms. However, the study carried out among youth corps trainees in Western province indicated that only 34% of the youth have ever used a condom, while 32% didn’t know where to go for a condom. The same study also reports that 51% of the youth did not want to use a condom in future sexual encounters [22].

In line with the previous study findings, age and sex appeared to have an impact on youth trainees’ knowledge of SRH. According to the present study findings, the youth trainee’s knowledge of SRH matters was positively correlated with the male gender where the male trainee’s knowledge of SRH was 2.3 times (95% CI:1.4–3.6) higher than the females. Further, the study results showed that the SRH knowledge of the trainees aged above 20 years was 1.9 times (95% CI:1.2–3.2) better than the younger trainees. Similar to the above finding a study that was carried out among Latin American youth indicated that the male gender and increasing age are associated with good sexual and reproductive health knowledge among youth [24]. It is obvious that SRH knowledge increases with older age and increased exposure to SRH information sources. Furthermore, in a country where sexuality remains a top-secret topic, and females are bound by certain restrictions prevailing in society, sources of information to females can be less than that of males. Nevertheless, in contrast to present findings, many previous pieces of research have shown that females are more knowledgeable on SRH matters than boys. A study done among Myanmar youth in 2013 showed that females were more likely to have a negative perception of premarital sex and more likely to be under the guardianship of their parents [25], which might prevent them from accessing sexual health information. However, the same study stated that although the males have more exposure to mass media compared to females, they had less exposure to reliable sources of SRH information [25]. However, the finding was against some studies that showed that girls are involved in sexual conversations to a greater extent than boys [26]. Another study also found that although parents tend to discuss sexual health information more with female children these discussions tend to be more cautious and focus mostly on the negative consequences of sexual activity [27]. Yet, all the above studies had been carried out in the South-Asian region where “sex” is a “hush-hush” topic in society. In a country like Sri Lanka, where there are a lot of stigmas associated with sex education children tend to go in search of sexual information from other sources such as books, magazines, and the internet. The present study’s findings can therefore be related to young men’s increased enthusiasm for exploring sexual information. A study done among a sample of unmarried youth in three districts in Sri Lanka indicated that a significantly higher percentage of male youth engage in sexual intercourse compared to females [23].

A similar study that was conducted among youth trainees attached to youth corps centers in the western province in 2019/20 showed that youth trainees had a fair knowledge of STIs and HIV. The same study revealed that the trainee’s STI knowledge was positively associated with age, education level, and wealth quintile. Similar to the present study this study also indicated a significant difference in knowledge across the ethnic and religious categories [22].

## Conclusions and recommendations

The present study was carried out to explore the effectiveness of introducing sexual health module into the youth training curriculum. The results of the study indicated that the introduction of the module has not been very successful in improving the trainee’s sexual health knowledge, while the trainee’s sexual health practices were at a substandard level. The results indicate the need to upgrade the SRH module that has been included in the youth trainee’s knowledge on contraception and STI prevention is not up to the expected level. The requirement for continuous training of the instructors to deliver the module in an interesting and effective way is a requirement. Besides, as a result of the frequent turnover of the training instructors, there is difficulty in conducting the module sessions in the same effective manner throughout. Hence, development of effective IEC material is a requirement. Global literature indicate that using youth friendly approaches such as establishing peer education programs, integrating mobile apps, using text messages to reach students, are some of the strategies that can be used to improve the quality of SRH education to youth [2]. Peer group education programs is a youth friendly approach that can easily be adopted to the present study setting. Nonetheless, effectiveness of some of the above strategies may largely depend on the needs and availability of resources of the population served. Hence, adaptation of some of these strategies to a socio-economically deprived context as the present study setting needs to be explored.

However, it is suggested to conduct qualitative research among training instructors as well as the youth trainees to discover the pitfalls in effective delivery of the SRH module.

Moreover, the literature indicate that a quality sexual health education curriculum aims at developing critical knowledge and skills needed to promote healthy behaviors and avoid risks. It will increase student’s ability to analyze family, peer, media influences that impact on health, to access valid and reliable health information, increase student’s ability to communicate with family, peers and teachers about sexual matters, improve their ability for decision making on sexual health, and take responsibility of their own health [3]. In order to ensure the quality of the curriculum, it is recommended to compare the existing curriculum using a health education curriculum analysis tool and make changes and modifications accordingly.

### Public health implications of the study findings

The findings of the study indicate that although the SRH module has been included into the youth training curriculum it has not been able to achieve a satisfactory improvement in SRH knowledge or practices in youth trainees. Furthermore, the findings show the poor level of contraceptive as well as STI prevention knowledge and practices among youth trainees.

It is required to identify the best ways to deliver sexual and reproductive health information to youth. Just incorporating the sexual and reproductive health module into the curriculum would not be sufficient to deliver the message to the audience, unless the correct techniques have been identified and adopted. Literature indicate that on-site service provision is a better way to increase youth accessibility to services [2]. Establishing condom availability programs withing the institution premises, on-campus STI screening services, mobile health clinics, tele health services are some of the approaches that can be used.

### Strengths and limitations

Conducting the study on youth attached to youth training institutes enabled the retrieval of information related to SRH knowledge and practices among both schooling and non-schooling youth which was a major strength of this study. The use of a self-administered questionnaire would have encouraged the youth to come up with more reliable data, considering the societal stigma around the study topic.

Carrying out the study in the institutional setting was one main limitation of this study since it may have contributed to misinformation. There was a possibility that the youth did not come up with true information due to fear and embarrassment. Moreover, failure to conduct a baseline assessment of youth trainee’s sexual health knowledge and practices prior to the incorporation of the SRH module was a pitfall.

### Future research

Research has to be carried out to explore the deficiencies in the content and delivery of the SRH module in youth training centers. Qualitative research among youth trainees as well as teacher instructors is suggested. Future research should aim at finding the most effective ways to deliver SRH information to youth in youth training centers and universities in Sri Lanka. Testing of pre and post interventional knowledge, attitudes, and practices of SRH in students would help to find the most effective way to deliver SRH module to youth.

### Patient-public involvement

In the designing stage of the research, focus group discussions were carried out with a sample of youth and their mothers in the community to explore the sexual and reproductive health needs of the youth in Sri Lanka. One of the major concerns of both young persons and their mothers was the lack of SRH knowledge among youth which needs to be improved. This directed us to explore the present knowledge on SRH among both schoolings and out-of-school youth and to identify the need to modify the content in the SRH module. Since youth training centers give shelter to both schooling and non-schooling youth.

The findings of the study will be disseminated to officials in the youth ministry to proceed with the curriculum modifications. Advocacy programs will be carried out targeting the youth ministry officials as well as the education ministry to prove the requirement of comprehensive sex education for children.

## Data Availability

The data that support the findings of this study are available on request from the corresponding Author. The data are not publicly available since it contains information that could compromise the privacy of the participants.
